# *QuickStats:* Percentage* of Adults Who Had a Severe Headache or Migraine in the Past 3 Months, by Sex and Age Group — National Health Interview Survey, United States, 2018^†^^,^^§^

**DOI:** 10.15585/mmwr.mm6912a8

**Published:** 2020-03-27

**Figure:**
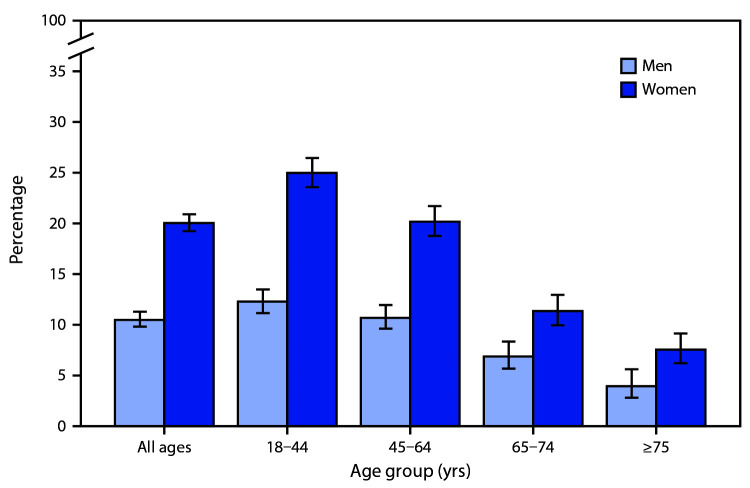
In 2018, women were nearly twice as likely as men to have had a severe headache or migraine in the past 3 months (20.1% versus 10.6%), both overall and within each age group. The percentage of persons experiencing severe headache or migraine declined with age for both men and women, from 25.5% among those aged 18–44 years to 7.6% among those aged ≥75 years for women and from 12.3% among those aged 18–44 years to 4.0% among those aged ≥75 years for men.

**Source:** National Health Interview Survey, 2018 data. https://www.cdc.gov/nchs/nhis.htm.

